# Effect of Accelerated Aging on Bamboo Fiber Lunch Box and Correlation with Soil Burial Degradation

**DOI:** 10.3390/polym14194220

**Published:** 2022-10-08

**Authors:** Huan Jiang, Ge Wang, Fuming Chen, Jianchao Deng, Xiaoyi Chen

**Affiliations:** 1 Department of Biomaterials, International Centre for Bamboo and Rattan, Beijing 100102, China; 2 Key Laboratory of National Forestry and Grassland Administration/Beijing for Bamboo & Rattan Science and Technology, Beijing 100102, China; 3 College of Material Science & Technology, Beijing Forestry University, Beijing 100083, China

**Keywords:** bamboo fiber lunch box, damp–heat treatment, freeze–thaw cycle, artificial weathering cycle

## Abstract

This study aimed to investigate the mechanical property decay that might occur during actual use and soil burial degradation of bamboo fiber lunch boxes. For this, the effects of three accelerated aging methods, namely damp–heat treatment, freeze–thaw cycle, and artificial weathering cycle, on the tensile strength, dynamic viscoelasticity, and chemical composition of bamboo fiber lunch boxes were compared, and a correlation of their mechanical property decay with soil burial degradation was established to obtain an acceleration factor (SAF) with aging time as a reference. The results showed that the mechanical properties of the bamboo fiber lunch box decreased to different degrees under the three accelerated methods, and the tensile strength decreased to less than 50% after 36 h of damp–heat treatment, 5 freeze–thaw cycles, and 11 artificial weathering cycles. However, after 10 days, the mechanical property of lunch box in soil degradation decreased by more than 50%. Infrared spectroscopy demonstrated rapid hemicellulose degradation during damp–heat treatment and freeze–thaw cycle, as well as a minor quantity of lignin, and a significant amount of lignin under artificial weathering cycle. With the freeze–thaw cycle and the artificial weathering cycle, the relative crystallinity dropped quickly, by 32.3% and 21.5%, respectively, but under damp–heat treatment, the crystallinity dropped barely, by 43.5%. The damage caused by the freeze–thaw cycle to the mechanical properties of bamboo fiber lunch boxes was greater than that by the damp–heat treatment and artificial weathering cycle. The fluctuation of SAF under freeze–thaw cycle was also more drastic. Compared to the artificial weathering cycle, the damp–heat treatment was more stable and reliable in predicting the decay law of soil burial degradation tensile strength of bamboo fiber lunch boxes.

## 1. Introduction

People are paying increasing attention to ecologically friendly and safe materials for lunch boxes because of the environment and human health issues produced by disposable plastic lunch boxes [1,2,3]. Biodegradable lunch boxes (including biodegradable plastic lunch boxes, paper lunch boxes, plant fiber-based lunch boxes, starch-based lunch boxes, and so forth) are gradually replacing disposable plastic lunch boxes due to their advantages of having an abundance of renewable raw material sources, low prices, and no pollution after degradation [4,5,6]. Plant fiber-based and synthetic polyester-based lunch boxes are currently the most popular products on the market [7].

Recent studies on lunch boxes made of plant fibers mainly focused on how the boxes were prepared [8,9,10] and degraded [11,12,13]. However, the actual circulation environment for lunch boxes might involve low-temperature exposure during cold chain transportation, microwave heating during consumption, and serving of boiling food. The temperature and moisture in these conditions might impact their performance in use. Plant fibers are made up of macromolecules, such as cellulose, hemicellulose, and lignin, which have a large number of hydrophilic groups on their cell wall structure and are particularly sensitive to changes in moisture and heat [14,15], as well as wet expansion and dry contraction [16]. Studies [17,18,19] demonstrated that when frozen water was present in the cracks and pores, fibers experienced bonding, degradation, and crack growth phenomena, and plant-fiber materials performed the worst at low temperatures. Moving from lower to higher temperatures caused microcracks to widen even further, which increased water absorption, significantly reduced mechanical characteristics, and ruptured the matrix by plasticization or delamination. Accelerated aging, which amplifies the impacts of moisture, temperature, and other conditions on the lunch boxes, is typically used to explore the intrinsic response of performance to solve the performance degradation of plant-fiber lunch box materials during actual usage. Understanding of the accelerated aging process contributed to affecting the use of materials and degradation behavior.

Furthermore, few studies have reported on the relationship between accelerated aging and soil burial degradation of plant fiber-based lunch boxes, and the related literature focused primarily on using accelerated indoor aging to predict the long-term service life of plant-fiber composites outdoors, and predicting their actual service life by developing prediction models [20,21]. Plant fiber composites have a long natural aging time, and the models developed so far fall into two categories: the first is based on existing empirical formulas [22] (residual strength model, aging kinetics model, stress relaxation model, and so on), and the second is the development of an indoor accelerated aging environment spectrum using the environmental equivalent method [23]. Additionally, it is more typical to determine the parameters that serve as performance indicators to calculate the acceleration factor using the gray theory [24] and to create a correlation between accelerated aging and normal aging.

Bamboo fiber lunch boxes are molded from steam explosion bamboo fiber mixed with a small amount of tapioca starch. They have the benefits of a dense fiber interweave structure, high stiffness, and good water and oil resistance. The response of physicochemical properties of bamboo fiber lunch boxes to accelerated aging has not been explored yet, and no definitive link with soil burial degradation has been established. In this study, three types of treatments, including damp–heat treatment, freeze–thaw cycle, and artificial weathering cycle, were applied to observe the changes in the mechanical properties, dynamic thermomechanical properties, and chemical composition of bamboo fiber lunch boxes. The study also aimed to establish a correlation of their mechanical properties decay with soil burial degradation so as to provide scientific theoretical support and testing methods for bamboo fiber lunch boxes in terms of process improvement, service life, and natural degradation rate assessment.

## 2. Materials and Methods

### 2.1. Materials

The bamboo fiber of the lunch box is the natural original bamboo fiber with a diameter of 0.03–0.4 mm and a length of 10–250 mm in 2–3 year old bamboo (*Bambusa pervariabilis* McClure × *Dendrocalamopsis daii Keng f.*) grown in Chongqing province, China. The natural bamboo fibers were steam-exploded and mixed with a small amount of tapioca starch to form pulp with a beating degree of 37 ± 2 °SR by coarse and fine grinding. Finally, the pulp was vacuum filtered and molded at a temperature of 140 °C for 3 min. The mass ratio of bamboo fiber to tapioca starch is 97:3. The function of starch is to fill the gap caused by uneven distribution of bamboo fiber during the molding process, and increase the leak-proofness and strength of the lunch box.

### 2.2. Methods of Accelerated Aging

1. The damp–heat treatment method referred to the national standard GB/T 22894-2008 “Paper and board-Accelerated ageing-Moist heat treatment at 80 °C and 65% relative humidity” [25]. The test temperature and humidity were adjusted as follows: temperature 98 °C ± 2 °C, humidity 95% ± 5% relative humidity. After 12, 24, 36, 48, and 60 h, the samples were taken, baked at 103 °C until they were absolutely dry, and placed under 0% humidity for 1 week.
2. The freeze–thaw cycle method referred to Ref. [26], and some of the test parameters were modified in this study to suit the lunch box material. First, the lunch box specimens were immersed in water at 20 °C for 3 h, then placed in a constant-temperature water bath at 98 °C for 3 h, followed by in an environment of −20 °C for 20 h, and finally heated in an oven at 60 °C for 3 h. The aforementioned steps were one cycle. After 1, 3, 5, 7, and 9 cycles, the samples were taken, baked at 103 °C until they were absolutely dry, and placed under 0% humidity for 1 week.
3. Artificial weathering cycle method referred to the national standard GB/T 16422.3-2014 “Plastic-Methods of exposure to laboratory light sources-Part 3: Fluorescent UV lamps” [27]. The lunch box specimens were put in an ultraviolet (UV) weathering test machine (IX-2130A, Guangdong Aisirui Instrument Technology Co., Ltd., Dongguan, China) equipped with two UVA-340 lamps (H358, Q-Lab Corporation, Westlake, OH, USA). A cycle was set for 8 h of UV light and 4 h of constant-temperature water bath heating at 60 °C. After 3, 7, 11, 15, and 19 cycles, the samples were taken, baked at 103 °C until they were absolutely dry, and placed under 0% humidity for 1 week.

### 2.3. Soil Burial Degradation

The soil burial degradation performance of the lunch boxes was determined by using method C “soil burial test” in the national standard GB/T 19275-2003 “Evaluation of the potential biodegradation and disintegration ability of materials under the action of specific microorganisms” [28]. The specific procedure was as follows: the tensile specimens were buried in loose soil 5 cm below the surface layer, removed at the corresponding time, and the surface soil was removed and baked until absolutely dry and set aside. The soil was collected from the field soil in Huimin area of Binzhou, Shandong Province, with relative humidity of 70%–90%, pH value of 7–8, and temperature of 22 °C–25 °C.

### 2.4. Mechanical Properties

The tensile strength of the lunch box specimens under soil burial degradation and different accelerated aging methods was tested by a universal mechanical testing machine (MTS Systems, Co., Ltd., Beijing, China) according to the national standard GB/T 1040.3-2006 “Plastic-Determination of tensile properties—Part 3: Test conditions for films and sheets” [29]. The specific parameters were: a dumbbell shape of 115 mm in length, 25 mm in width and about 0.6 mm in thickness, and a stretching speed of 2 mm/min. Each group of samples was tested five times in parallel and the average value was taken.

### 2.5. Dynamic Thermomechanical Properties

The dynamic viscoelastic properties of the lunch boxes was tested in the tensile mode of the DMA Q800 (TA Instruments, Camden, NJ, USA). The instrumental conditions were: amplitude 8 μm, frequency 1 Hz, heating rate 2 °C/min, temperature range 40 °C–300 °C. The sample size was 35 × 5 × 0.6 mm. Before the test, all specimens were in an adiabatic state.

### 2.6. Chemical Group

The samples in different accelerated aging methods were dried and ground with potassium bromide at a ratio of 1:100. The chemical group was observed using Fourier transform infrared (FTIR) spectroscopy (VERTEX 80 V, Bruker, Karlsruhe, Germany). The wavelength ranged from 4000 to 400 cm^−1^, and the number of scans was 64.

### 2.7. Relative Crystallinity

Determining crystallinity is an approach to understanding the effect of weathering on wooden materials [30]. Dried 60-mesh specimen powder was taken and scanned in the reflection mode of an X-ray diffractometer (Bruker, Karlsruhe, Germany) to test its crystallinity. The parameters of the test conditions were: scanning speed 5°/min; step size 0.02°; scanning range 5–60°; voltage 40 kV; and current 40 mA. The relative crystallinity was calculated according to Segal’s empirical method (Equation (1)) [31].

(1) $$CrI\%=\left(I_{002}-I_{am}\right)/I_{002}$$

## 3. Results and Discussion

### 3.1. Mechanical Properties

**Figure 1:**
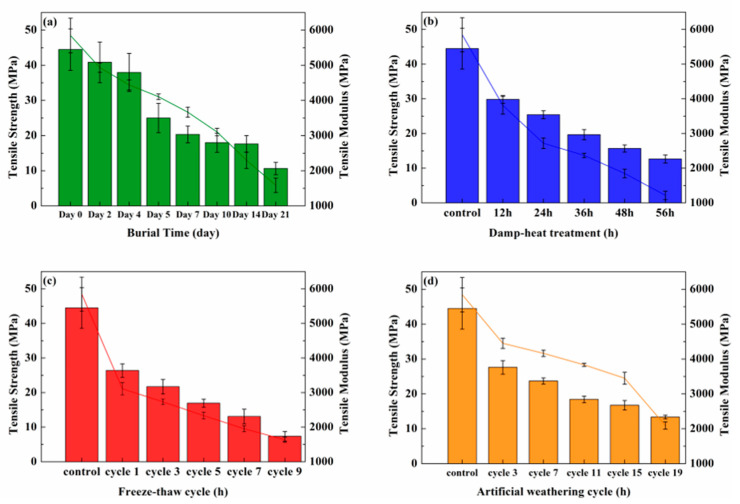
Tensile strength and modulus: (**a**) soil burial degradation; (**b**) damp–heat treatment; (**c**) freeze–thaw cycle; and (**d**) artificial weathering cycle.

Figure 1 depicts the effects of damp–heat treatment, freeze–thaw cycle, and artificial weathering cycle on the tensile strength and modulus of bamboo fiber lunch boxes. Under damp–heat treatment, the modulus dropped from 5845 MPa to 1217 MPa, while the strength dropped from 44.7 MPa to 12.6 MPa. The reason was the diffusion of a large number of water molecules into the interior of the lunch box under high temperature and high humidity, and the combination of water molecules and a large number of free hydroxyl groups in the bamboo fiber [32,33], resulting in the destruction of hydrogen bonds and the resolution of the fiber matrix. The pores were enlarged, and microcracks were generated, expanded, and interconnected, resulting in the reduction in the mechanical properties of the lunch box [34].

The modulus decreased from 5845 MPa to 1633 MPa, while the strength decreased from 44.7 MPa to 7.4 MPa under freeze–thaw cycle, which showed that the maximum decrease was about 80%. The reason was [26,35] that when soaking at room temperature, water entered the voids in the fiber and large pores, jointed filaments, and starch granules already in the molding process, which reduced the bonding strength. In a constant-temperature water bath, the effect of moisture and high temperature made the layers of molded fibers gradually defective and separated. In the low-temperature freezing process, frozen water was used on the fibers to produce pressure. In drying, the water in the pores was thawed and then absorbed by the fine pores. Under the alternation of positive and negative temperatures and the flow and absorption of hot and cold water, the dense interwoven structure of the lunch box was destroyed, the number of voids increased, the volume increased, and fractures easily occurred, thus gradually reducing the mechanical properties [36].

Under artificial weathering cycle, the modulus decreased from 5845 MPa to 2094 MPa, while the strength decreased from 44.7 MPa to 13.4 MPa. In the early stage, UV light could cause surface yellowing and darkening, increased surface roughness, and small cracks appeared, thus reducing its mechanical strength. The reason was the formation of free radicals from reactive groups in lignin, such as secondary alcohols, hydroxyl, carboxyl, aromatic, and phenolic groups, after the irradiation of the lunch box by 340 nm UV light, and a large number of free radicals combined with oxygen and eventually formed quinone structures through intermediates [37]. In addition, hot steam could cause erosion that further accelerated the photodegradation process, where cracks gradually expanded into the interior of the bamboo lunch box [38]. Light continuously reflected and refracted within the cracks, which initiated additional photodegradation on the newly exposed surfaces inside the materials.

### 3.2. Dynamic Thermomechanical Properties

**Figure 2:**
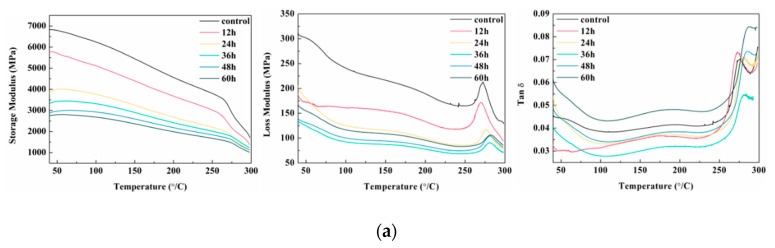

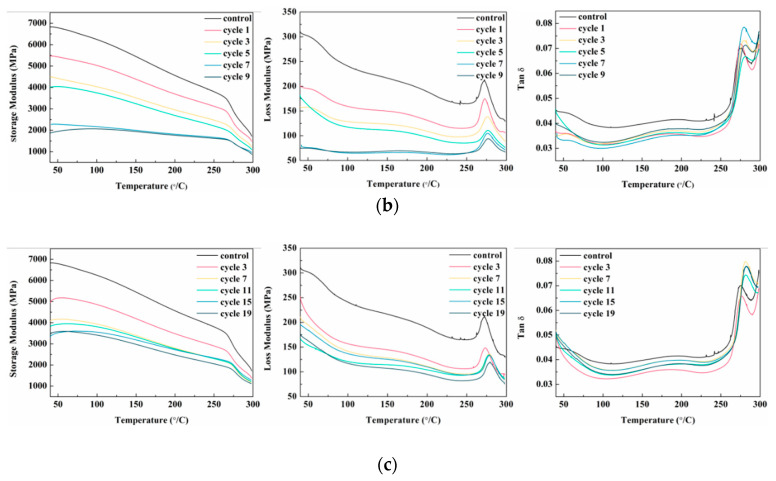
Dynamic thermomechanical properties: (**a**) from left to right, E′, E″, tan*δ* under damp–heat treatment; (**b**) from left to right, E′, E″, tan*δ* under freeze–thaw cycle; and (**c**) from left to right, E′, E″, tan*δ* under artificial weathering cycle.

Figure 2 shows the dynamic thermomechanical properties of the lunch boxes under damp–heat treatment, freeze–thaw cycle, and artificial weathering cycle. In the whole test temperature range [39,40,41], E′ decreased continuously as the temperature increased, and E′ decreased slowly before approaching the glass transition temperature (T_g_). At this time, the molecular chain segments were in a frozen state, only a small part of the side groups and bond angles moved, the deformation was tiny, and the modulus value remained in a large range. After T_g_, the molecular chain segments moved strongly, the crystalline region was destroyed, the deformation became larger, and E′ decreased rapidly. As the temperature increased, E″ first increased and then decreased, with a smaller loss peak at 70 °C–90 °C formed by the relaxation of the action of polar groups in the hemicellulose side chain, also known as β relaxation. Near T_g_, E″ reached a peak, called α relaxation, which was caused by the glass transition of hemicellulose and lignin.

As the time of damp–heat treatment increased, E′ kept decreasing, which was caused by increasing molecular chain breaks and decreasing fiber strength due to thermal softening and decomposition of polysaccharides. The reason was that the hydrogen bonding between the molecules of bamboo fibers was weakened by the entry of water molecules and the large number of hydroxyl groups in bamboo fibers at first, which resulted in an increase in the intermolecular spacing and weakening of bonding energy, and an increase in the ductility of molecular chains, showing that the curve moved toward the low temperature. Later, under the action of high temperature and water, hemicellulose and other non-crystalline substances precipitated out, while cellulose reacted with water, molecular chains broke, and fibers were easily separated from the molded fracture interface, which showed a curve shift toward high temperature [42]. The main transition peak of tan*δ* was consistent with E″, and the height of the secondary transition peak gradually decreased, indicating that the structure of the lunch box matrix was destroyed and the molecular chain movement was weakened at the later stage of moist heat aging.

As the number of freeze–thaw cycles increased, E′ decreased continuously because water molecules entered the amorphous area of bamboo fibers to make the fibers swell. The hemicellulose degradation and lignin precipitated on the surface of the lunch box after the steam burst was dissolved, resulting in a lower degree of cross-linking, which reflected in lower E′ and increased plasticity [43]. In E″, the temperature near T_g_ moved to high temperature with the increase in freeze–thaw cycles. The main transition of cellulose occurred around 200–250 °C, while the main transition of amorphous substances, such as lignin and hemicellulose, occurred below 200 °C [44] because the hemicellulose and lignin decreased and the cellulose content relatively increased, resulting in the shift to high temperature. The main transition peak in tan*δ* shifted toward high temperature, and the height of the secondary transition peak decreased, indicating that the molecular activity decreased and the amorphous substances due to easy moisture absorption and degradation decreased.

The continuous photolysis of lignin and the decrease in mechanical strength caused a constant decrease in E′. The peak near T_g_ shifted toward higher temperatures in E″, which was also caused by a decrease in lignin and a relative increase in the cellulose content with a higher primary transition temperature than lignin [45]. Similarly, in tan*δ*, the primary transition peak was consistent with E″, and the secondary transition peak gradually decreased in height.

### 3.3. Fourier Transform Infrared Spectroscopy

**Figure 3:**
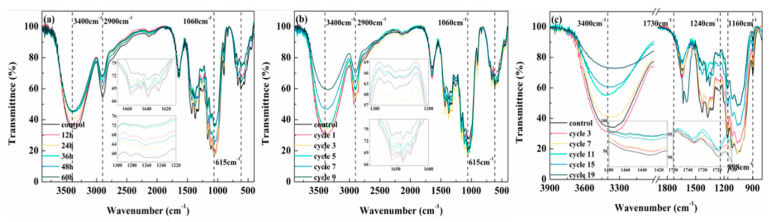
FTIR: (**a**) damp−heat treatment; (**b**) freeze−thaw cycle; and (**c**) artificial weathering cycle.

Figure 3 displays the infrared spectra of lunch boxes after being exposed to damp–heat treatment, freeze–thaw cycle, and artificial weathering cycle. Freeze–thaw cycle and damp–heat treatment resulted in the peak intensity at 3400 cm^−1^ being slightly lower, which was attributed to the two free hydroxyl groups in the cellulose amorphous region combined at a high temperature and the formation of hydrogen bonds by the action of water molecules. In addition, the peak intensity at 2900 cm^−1^ and 1060 cm^−1^ reduced, owing to the decomposition of aliphatic, β-glycoside structure and other substances [26,45]. Degradation of lignin was manifested in a decrease in the peak at 1640 cm^−1^ and 1260 cm^−1^, which was attributed to the benzene ring skeleton vibration [46,47]. Under the artificial weathering cycle [48,49], the intensity of O-H stretching vibration at 3400 cm^−1^ decreased; the C-O stretching vibration representing lignin at 1240 cm^−1^, the aromatic skeleton vibration of lignin at 1420 cm^−1^, and the C-H bending vibration of lignin at 1457 cm^−1^ all kept decreasing with the number of cycles, indicating that the composition of lignin was decreasing. Meanwhile, the C-O-C stretching vibration of hemicellulose at 1160 cm^−1^ and the C-H bending vibration intensity of cellulose at 898 cm^−1^ both significantly reduced. The C=O stretching vibration of hemicellulose at 1730 cm^−1^ reduced, indicating that the UV light absorbed by bamboo fiber lunch boxes reacted with the phenolic hydroxyl group of lignin to form free radicals, which were subsequently transformed into unsaturated carbonyl compounds via demethylation or side-chain cleavage.

### 3.4. Relative Crystallinity

**Figure 4:**
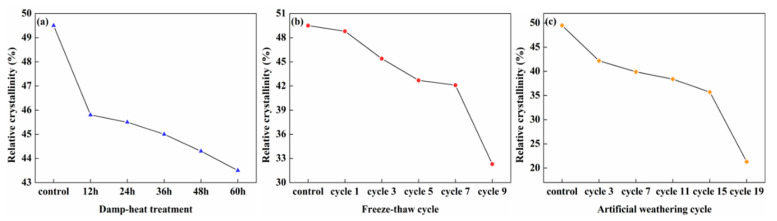
Relative crystallinity: (**a**) damp–heat treatment; (**b**) freeze–thaw cycle; and (**c**) artificial weathering cycle.

According to Figure 4, the relative crystallinity of the lunch boxes decreased to different degrees after damp–heat treatment, freeze–thaw cycle, and artificial weathering cycle treatments. Under damp–heat treatment, the relative crystallinity was from 49.5%, to 43.5%. Under freeze–thaw cycles, the relative crystallinity of the lunch boxes was from 49.5% to 32.3%. Under the artificial weathering cycle, the relative crystallinity was from 49.5% to 21.5%. First, the main chemical components in the bamboo fiber lunch box exhibited different degrees of degradation, including a certain degree of hydrolysis and pyrolysis of cellulose components under the action of moisture and steam, precipitation, and hydrolysis of hemicellulose and lignin components on the surface of the lunch box after a steam burst. Second, the continuous heat–freeze–thaw led to the migration of water in the interwoven structure of bamboo fibers, which damaged the crystalline area of cellulose to a certain extent. Finally, the lignin was continuously photolyzed, and the hot and humid steam entered the void of the lunch box, causing the deepening of photolysis.

### 3.5. Comparison of Accelerated Aging Test Results

#### 3.5.1. Comparison of Correlation Analysis of Tensile Strength of Lunch Boxes after Accelerated Aging

The tensile strength and modulus after accelerated aging using the three methods were correlated by SPSS software (Version 22.0. IBM Corp., Armonk, NY, USA), and the data were analyzed by Pearson correlation analysis. Table 1 shows that the correlation of the tensile strength and modulus of the lunch boxes after damp–heat treatment, freeze–thaw cycle, and artificial weathering cycle was positive and highly correlated at 0.01 and 0.05 significant levels.

#### 3.5.2. Correlation Analysis of Accelerated Aging and Soil Burial Degradation

On the basis of the aforementioned three sets of data showing high correlation, regression analysis of the tensile strength and modulus of the lunch boxes was carried out by SPSS software to simulate the changes in the main mechanical properties of the lunch boxes at different stages of the accelerated aging treatment process. According to the more typical life model in the accelerated aging test and the actual situation, linear and exponential functions were chosen to be used for the regression analysis. The data were fitted using the curve parameter estimation method with the aging cycle stage as the independent variable and tensile strength and modulus as the dependent variable. Table 2 shows the curve fitting results of the tensile strength and modulus of the lunch boxes under soil burial degradation, damp–heat treatment, freeze–thaw cycle, and artificial weathering cycle and their significance test results. The results given in Table 2 are significant under both linear and exponential functions.

Among them, the exponential function fitted significantly better than the linear function, while the tensile strength fitted more significantly than the tensile modulus. The exponential function was chosen as the final fit function for the computational analysis of the acceleration factor.

**Figure 5:**
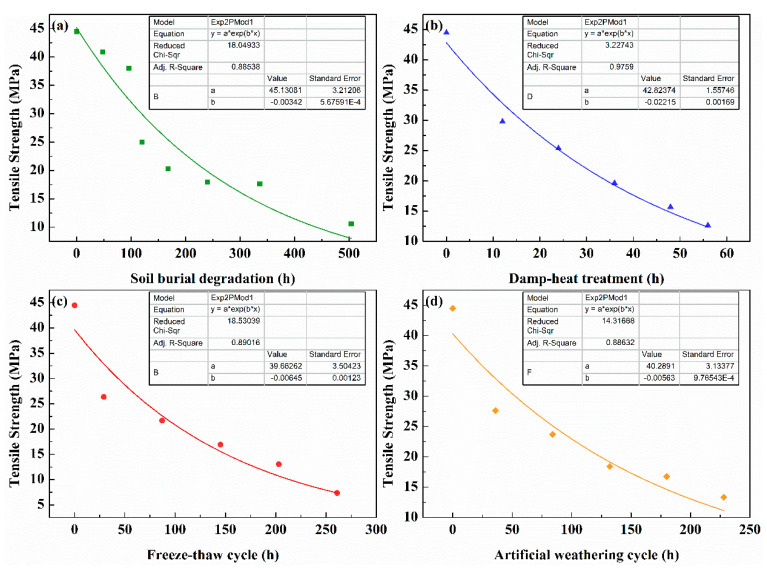
Fitting curves for different aging methods of bamboo fiber lunch boxes (**a**) soil burial degradation; (**b**) damp–heat treatment; (**c**) freeze–thaw cycle; and (**d**) artificial weathering cycle.

Figure 5 shows the tensile strength fitting results for the three accelerated aging methods and soil burial degradation obtained using origin, and the fitting equations were:

y = 45.13081 × exp(−0.00342 × x)(2)

y = 42.82374 × exp(−0.02215 × x)(3)

y = 39.66262 × exp(−0.00645 × x)(4)

y = 40.2891 × exp(−0.00563 × x)(5)

Figure 5 shows that the *R*^2^ of the fitted equations was in good agreement: soil burial degradation: 0.88538; damp–heat treatment: 0.9759; freeze–thaw cycle: 0.89016; and artificial weathering cycle: 0.88632. Using the fitted Equations (2)–(5), the time required for soil burial degradation and accelerated aging, when the tensile strength of the lunch box decreased to the same value as during aging, as well as the calculated acceleration factor (SAF) [50] could be evaluated, and the obtained results are given in Table 3.

From the fitting results, the soil burial degradation time was 172 h when the strength of the bamboo fiber lunch box was 25 MPa. In comparison, accelerated aging only required 24 h, 71 h, and 84 h, and the acceleration multipliers were 7.1, 2.4, and 2.0 times, respectively. The multiplication rate under these three accelerated methods decreased continuously with increasing time. For example, the tensile strength was 10 MPa at 440 h of soil burial degradation, and the acceleration multipliers of indoor accelerated aging experiments were 6.7, 2.0, and 1.7, respectively. The acceleration factors were slowly stabilized and no longer fluctuated greatly as the tensile strength kept decreasing in the later period.

Table 3 shows that the fluctuation of SAF by freeze–thaw cycle was larger compared with that by damp–heat treatment and artificial weathering cycle, which was due to the larger damage of freeze–thaw cycle to the bamboo fiber lunch boxes. The fluctuation of acceleration factor with aging time as a reference was larger mainly because the temperature and humidity were constant during accelerated aging; however, various factors, such as microorganisms and moisture, were involved in the decomposition of the lunch boxes during soil burial degradation. The SAF fluctuated less, which was more stable and reliable in predicting the strength decay, among which, the fluctuation of SAF in the damp–heat treatment was slightly less than that in the artificial weathering cycle.

## 4. Conclusions

Bamboo fiber lunch boxes had the advantages of a plentiful supply of raw materials, high stiffness, and ease of degradation. In this study, three accelerated aging methods were used to study the mechanical decay of bamboo fiber lunch boxes, and established tensile strength as an indication for the relationship between accelerated aging and soil burial deterioration. The main conclusions of this study were as follows:

1. Freeze–thaw cycle had the most severe effects, with the greatest impact on lunch box performance degradation. It showed 7.4 MPa strength and 32.3% relative crystallinity at 9 cycles, a significant decrease in cellulose and hemicellulose content. The strength and crystallinity of damp–heat treatment were 12.6 MPa and 43.5% after 56 h. The lignin of the lunch box under artificial weathering cycle underwent massive decomposition, and the strength and relative crystallinity were 13.4 MPa and 21.5% after 19 cycles.
2. When establishing the correlation between accelerated ageing and soil burial degradation, the tensile modulus is not as significant as the tensile strength. The damp–heat treatment was used to estimate the decay law of soil burial degradation tensile strength of the bamboo fiber lunch boxes and was more stable and reliable than the SAF fluctuation of freeze–thaw cycle and artificial weathering cycle. Additionally, the generalized calculation of the aging acceleration factor based on aging time was restricted. In the study of accelerated aging of meal boxes, acceleration factors, such as irradiation, can be used as a reference for prediction, and these merit further investigation in the future.

## Figures and Tables

**Table 1:** Tensile strength correlation coefficients under different aging methods.

**Descriptive Statistics**						
		Mean		Std. Deviation		N
Soil burial degradation	Strength	26.8488		12.54872		8
	Modulus	3747.6625		1394.17945		8
Damp–heat treatment	Strength	24.6033		11.57316		6
	Modulus	2968.0350		1657.44982		6
Freeze–thaw cycle	Strength	21.6417		12.98841		6
	Modulus	2933.4467		1521.02674		6
Artificial weathering cycle	Strength	24.0417		11.22202		6
	Modulus	3972.1533		1232.36292		6
**Correlations**						
			Soil burial degradation	Damp–heat treatment	Freeze–thaw cycle	Artificial weathering cycle
Soil burial degradation	Pearson Correlation	Strength	1	0.920 **	0.894 *	0.876 *
		Modulus	1	0.983 **	0.936 **	0.980 **
	Sig.(2-tailed)	Strength		0.009	0.016	0.022
		Modulus		0.000	0.006	0.001
	Sums of Squares and Cross-products	Strength	1102.293	605.105	660.240	558.926
		Modulus	13,606,154.39	7,871,195.294	6,879,026.470	5,833,421.266
	Covariance	Strength	157.470	121.021	132.048	111.785
		Modulus	1,943,736.341	1,574,239.059	1,375,805.294	1,166,684.253
	N	Strength	8	6	6	6
		Modulus	8	6	6	6
Damp–heat treatment	Pearson Correlation	Strength	0.920 **	1	0.997 **	0.994 **
		Modulus	0.983 **	1	0.977 **	0.950 **
	Sig.(2-tailed)	Strength	0.009		0.000	0.000
		Modulus	0.000		0.001	0.004
	Sums of Squares and Cross-products	Strength	605.105	669.691	749.011	645.206
		Modulus	7,871,195.294	13,735,699.58	12,314,439.88	9,704,258.728
	Covariance	Strength	121.021	133.938	149.802	129.041
		Modulus	1,574,239.059	2,747,139.916	2,462,887.976	1,940,851.746
	N	Strength	6	6	6	6
		Modulus	6	6	6	6
Freeze–thaw cycle	Pearson Correlation	Strength	0.894 *	0.997 **	1	0.995 **
		Modulus	0.936 **	0.977 **	1	0.911 *
	Sig.(2-tailed)	Strength	0.016	0.000		0.000
		Modulus	0.006	0.001		0.012
	Sums of Squares and Cross-products	Strength	660.240	749.011	843.493	725.250
		Modulus	6,879,026.470	12,314,439.88	11,567,611.77	8,539,016.058
	Covariance	Strength	132.048	149.802	168.699	145.050
		Modulus	1,375,805.294	2,462,887.976	2,313,522.353	1,707,803.212
	N	Strength	6	6	6	6
		Modulus	6	6	6	6
Artificial weathering cycle	Pearson Correlation	Strength	0.876 *	0.994 **	0.995 **	1
		Modulus	0.980 **	0.950 **	0.911 **	1
	Sig.(2-tailed)	Strength	0.022	0.000	0.000	
		Modulus	0.001	0.004	0.012	
	Sums of Squares and Cross-products	Strength	558.926	645.206	725.250	629.668
		Modulus	5,833,421.266	9,704,258.728	8,539,016.058	7,593,591.892
	Covariance	Strength	111.785	129.041	145.050	125.934
		Modulus	1,166,684.253	1,940,851.746	1,707,803.212	1,518,718.378
	N	Strength	6	6	6	6
		Modulus	6	6	6	6

**: correlation significant at 0.01; *: correlation significant at 0.05.

**Table 2:** Curve estimation results and significance test of tensile strength after soil burial degradation.

Fitted Objects		Fitting Type	Mean Square	Df	R^2^	F	Sig.F
Soil degradation	Strength	Linear	874.166	1	0.793	22.992	0.003
			38.021	6			
		Exponential	1.567	1	0.900	53.714	0.000
			0.029	6			
	Modulus	Linear	12,904,038.14	1	0.948	110.273	0.000
			117,019.374	6			
		Exponential	1.278	1	0.997	2150.041	0.000
			0.001	6			
Damp–heat treatment	Strength	Linear	623.422	1	0.931	53.896	0.002
			11.567	4			
		Exponential	1.027	1	0.988	331.036	0.000
			0.003	4			
	Modulus	Linear	12,499,537.15	1	0.910	40.446	0.003
			309,040.606	4			
		Exponential	1.460	1	0.970	128.712	0.000
			0.011	4			
Freeze–thaw cycle	Strength	Linear	707.240	1	0.838	20.762	0.010
			34.063	4			
		Exponential	1.806	1	0.950	76.528	0.001
			0.024	4			
	Modulus	Linear	7,966,935.369	1	0.689	8.850	0.041
			900,169.100	4			
		Exponential	0.840	1	0.844	21.571	0.010
			0.039	4			
Artificial weathering cycle	Strength	Linear	515.231	1	0.818	18.003	0.013
			28.609	4			
		Exponential	0.845	1	0.931	53.719	0.002
			0.016	4			
	Modulus	Linear	6,861,510.934	1	0.904	37.490	0.004
			183,020.239	4			
		Exponential	0.509	1	0.874	27.665	0.006
			0.018	4			

**Table 3:** Relationship between soil burial degradation and accelerated aging methods.

Tensile Strength /MPa	Aging Time/h				SAF		
	Soil Degradation	Damp–Heat Treatment	Freeze–Thaw Cycle	Artificial Weathering Cycle	Damp–Heat Treatment	Freeze–Thaw Cycle	Artificial Weathering Cycle
30	119.4	16.1	43.3	52.4	7.4	2.7	2.3
25	172.7	24.3	71.6	84.7	7.1	2.4	2.0
20	237.9	34.4	106.2	124.4	6.9	2.2	1.9
15	322.1	47.4	150.7	175.5	6.8	2.1	1.8
10	440.6	65.6	213.6	247.5	6.7	2.0	1.7
5	643.3	96.9	321.1	370.6	6.6	2.0	1.7

## Data Availability

All results from the data analysis needed to evaluate this report are available in the main text or in the tables and figures.
